# Antioxidant, Antimicrobial Activity and Total Phenolic Content within the Aerial Parts of *Artemisia* *absinthum, Artemisia santonicum *and* Saponaria officinalis*

**Published:** 2011

**Authors:** Memnune Sengul, Sezai Ercisli, Hilal Yildiz, Neva Gungor, Arzu Kavaz, Bülent Çetin

**Affiliations:** a Department of Food Engineering, Faculty of Agriculture, Ataturk University, 2524 Erzurum, Turkey.; b Department of Horticulture, Faculty of Agriculture, Ataturk University, 25240 Erzurum, Turkey.; c Department of Nutrition and Dietetics, Health School, Erzincan University, 24100 Erzincan, Turkey.

**Keywords:** *Artemisia*, Antioxidants, Antibacterial activity, *Saponaria*

## Abstract

Three native Turkish medicinal and aromatic plants *(Artemisia absinthum, Artemisia santonicum and Saponaria officinalis)* were investigated to analyze their antioxidant activity, total phenolic content and antimicrobial activity. Their total antioxidant activity was determined by using a β-carotene bleaching assay and their antimicrobial activity was determined by utilizing an agar disc diffusion assay. Methanol extracts of the three species analyzed showed high antioxidant activity and among them *Artemisia absinthum* possessed the highest quantity (71.78%). The total phenolic content (Folin-Ciocalteu assay) was shown to be between 6.57 μgGAE/mg dry weight basis *(Saponaria officinalis)* and 8.86 μgGAE/mg dry weight basis *(Artemisia absinthum)*. There was a positive correlation (R = 0.819) between the total phenolic content and the antioxidant activity measured in the plant samples. The aqueous and methanol extracts of the aerial parts of the species showed antibacterial activities against a number of microorganisms. The methanol extracts were found to inhibit the growth of microorganisms more than the aqueous extracts. These findings suggest that the methanol extracts of the plants tested contain compounds with antimicrobial properties. These exhibited properties propose that such plant extracts can possibly be used as natural preservatives in the food and pharmaceutical industries.

## Introduction

Herbal medicine represents one of the most important aspects of traditional medicine in Turkey, especially in rural areas. As such, phytotherapy is practiced by a large proportion of the Turkish population for treating several physical, physiological, mental and social ailments (1). To promote the proper use of herbal medicine and to determine their potential as new drugs sources it is essential to study medicinal plants. In particular plants that have been well known for their use in folklore medicine (2). Plants are known to produce certain bioactive molecules which react with other organisms in the environment and in turn cause the inhibition of bacterial or fungal growth (antimicrobial activity). Medicinal plants that have been traditionally used tend to produce a variety of compounds with known therapeutic properties (3, 4). Substances that can inhibit the growth of pathogens and exhibit only slight toxicity on host cells are considered as being good candidates for the development of new antimicrobial drugs. 

Spices and herbs have been used for thousands of centuries in many cultures to enhance the flavor and aroma of foods. Scientific experiments since the late 19th century have documented the various antimicrobial properties of certain spices, herbs, and their components (5). Many studies have reported the activities of spices and herbs on food borne pathogenic bacteria (6, 7).

The genus *Artemisia *consists of small herbs and shrubs. This genus is one of the largest and most widely distributed genera of the Compositae or Asteraceae family (1-8). Members of this genus are of botanical and pharmaceutical interest due to their characteristic scent or taste, and are used in the liqueur-making industry (1-9). *Artemisia *species have been used in folk remedies as an antipyretic, antiseptic, antihelminitic, tonic, diuretic and for the treatment of stomach ache (1). There are about 22 species of the genus *Artemisia *found in Turkey (8). Among them *Artemisia absinthium, *known locally as pelin otu, grows naturally in wide regions of Anatolia. *Artemisia santonicum, *which is known as Yavşan, grows on sandy spots and salted lands in Turkey. This species has been used in the treatment of diabetes (1). The anti-diabetic properties of this plant have been confirmed (10).

*Saponaria officinalis, *which is known as Coven, is a herbaceous perennial, 30-100 cm tall. The plant grows in moist ditches, along roadsides, waste places, near old home sites, in meadows, and as a planted ornamental. *Saponaria officinalis *contains large amounts of saponins, which froth when extracted using water. *Saponaria officinalis *has been used as an alternative medicine since the time of Dioscorides. Its medicinal uses include it being used as an antiscrophulatic, cholagogue, depurative, diaphoretic, mildly diuretic, expectorant, purgative and tonic. A decoction of the herb is applied externally to treat itchy skin (1).

Although *Artemisia absinthum, Artemisia santonicum *and *Saponaria officinalis *have been used as folk remedies to treat various ailments in Turkish folk medicine (1), as yet there has been little attempts made to study the antioxidant and antimicrobial potential of these plants against a wide range of food-associated microorganisms. 

As such the aims of this study were to estimate the total phenolic content, antioxidant activity and antimicrobial activity of *Artemisia absinthum, Artemisia santonicum *and *Saponaria officinalis*.

## Experimental

*Plant materials*

Aerial parts (leaves, flowers, and stems) of plants (*Artemisia absinthum, Artemisia santonicum and Saponaria officinalis*) were collected at the end of July in 2007 from Erzurum located in the Eastern Anatolia region of Turkey. The samples were then dried at 50^o^C in an oven and ground to form a fine powder using a mortar and pestle. The resulting powder was then kept at room temperature prior to extraction for analyzing its antioxidant activity, total phenolic content and antimicrobial activity.

*Extraction of plant material for total phenolic content and antioxidant activity*

10 mg of the grounded sample was mixed with 10 mL methanol and then stirred for 30 min using a magnetic stirrer**. **The suspension was subsequently filtered through Whatman No. l filter paper. The resulting final solutions were used as a stock solution for analyzing the total phenolic content and antioxidant activity (11).

*Determination of total phenolic content*

The concentration of total phenolics in the methanol extract of *Artemisia absinthum, Artemisia santonicum *and *Saponaria officinalis *was determined by the Folin–Ciocalteau colorimetric method (12). Briefly, one ml of the solution (containing 1mg) extract in ethanol was pipetted into a flask. Then 46 mL of distilled water and 1 mL of Folin-Ciocalteu’s reagent was added and mixed thoroughly. The mixture was left to stand for 3 min to which 3 mL of 2% sodium carbonate was then added. After 120 min of incubation at ambient temperature with constant shaking, the resulting absorbance was measured at 760 nm. Measurements were carried out in duplicate and a calibration curve was formed using gallic acid. The results were expressed as μgGAE/mg.

*Determination of antioxidant activity*

The antioxidant activity of the methanol extracts of *Artemisia absinthum, Artemisia santonicum *and *Saponaria officinalis *was determined according to the *β*-carotene bleaching method described by Kaur and Kapoor (13). Briefly, 4 mL of *β*-carotene solution (0.1 mg in 1 mL chloroform), 40 mg of linoleic acid and 400 mg of Tween 40 were transferred to a round-bottom flask. The mixture was then evaporated at 50°C by means of a rotary evaporator to remove the chloroform present. Afterwards 100 mL of oxygenated distilled water was added slowly to the residue and vigorously agitated to achieve a stable emulsion. Subsequently 800 μL of the extracts were added to 3 mL aliquots of the *β*-carotene/linoleic acid emulsion. As soon as the emulsion was added to each tube, the time zero absorbance was measured at 470 nm using a spectrophotometer. The mixtures were incubated at 50°C for a period of 90 min. Measurements were taken at 15 min intervals for 90 min. Methanol was used as the control. A blank, devoid of *β*-carotene**, **was prepared for background subtraction**. **BHA and BHT were used as the standard. All samples were assayed in triplicate. The degradation rate (DR) was calculated according to first order kinetics, using the following equation based on:

ln (*a/b*) x 1*/t *= DR_sample _or Dr_standart_

Where ln is the natural log, *a *is the initial absorbance (470 nm) at time 0, *b *is the absorbance (470 nm) at 100 min and *t *is the time. The antioxidant activity (AA) was expressed as percent of inhibition relative to the control, using the following formula:

AA = (DR_control_ - DR_sample_ or _standart_/DR_control_) × 100

*Determination of antimicrobial activity*

*Test organisms*

Methanol and aqueous extracts of the samples were individually tested against a range of 26 microorganisms, including 21 bacteria, 3 fungi and 2 yeast species. To analyze the antimicrobial activity of the extracts the following microorganisms were used: *Bacillus subtilis, Staphylococcus aureus, Streptococcus pyogenes, Salmonella typmiruim, Saccharomyces cereviciae, Bacillus cereus, Candida albicans, Streptococcus thermophilus, Pseudomonas aeruginosa, Klebsiella pneumonia *subsp. *Pneumonia, Staphylococcus hominis, Enterobacter cloaceae, Escherichia coli, Proteus mirabilis, Klebsiella pneumonia *subsp*. Ozanae, Providencia alcaliaciens, Acinetobacter lwoffi, Pseudomonas pseudoalcaligenes, Pseudomonas fluorescens, Pseudomonas putida, Flavobacterium indologenes, Yersinia enterocolitica, Alcaligenes feacalis, Penicillium brevicompactum, Trichothecium roseum *and *Aspergillus niger.*

The bacteria, maintained on nutrient agar (Merck, Darmstadt, Germany) were supplied by the microbiology laboratory of the Agricultural Faculty of Ataturk University, Erzurum, Turkey. The identity of the bacteria used in this study was confirmed using the microbial identification system in the biotechnology application and research center at ataturk university.

*Extraction of plant material for antimicrobial activity*

The dried and powdered herb materials (400 g) were extracted successively in a Soxhlet using methanol (MeOH) for 72 h at a temperature not exceeding the boiling point of the solvent (14). The extracts were filtered using Whatman filter paper (No : 1) and then concentrated in vacuo at 40°C using a rotary evaporator. For aqueous extracts, 5 g of each spice was soaked in 95 mL distilled water for 1 h at room temperature with occasional stirring followed by gentle boiling for 2 min on a plate heater equipped with a magnetic stirrer (Are2, VELPR, Italy). The extract was obtained by cooling and filtering through Whatman No 1 filter paper. The residues obtained were stored in a freezer at −80°C until use (15).

*Disc-diffusion assay*

The extracts were dissolved in the same solvent (methanol and water) to achieve a final concentration of 30 mg/mL and afterwards sterilized by filtering through 0.45 μm Millipore filters (Schleicher and Schuell, Microscience, Dassel, Germany). Antimicrobial tests were then carried out by disc diffusion using 0.1 mL of suspension containing 10^8^ CFU/mL of bacteria, 10^6^ CFU/mL of yeast and 10^4 ^spore/mL of fungi spread on nutrient agar (NA), sabouraud dextrose agar (SDA), and potato dextrose agar (PDA) medium, respectively. The discs (6 mm in diameter) were impregnated with 30 mg/ mL extracts (300 μg/disc) and placed on the inoculated agar. Negative controls were prepared using the same solvents employed to dissolve the plant extracts. SCF105 (30 μg sulbactam + 75 μg cefoperazona/disc), NV30 (30 μg novobiocin/ disc), NV5 (5 μg novobiocin/disc), SAM20 (10 μg sulbactam + 10 μg ampicillin/disc), CC2 (2 μg clindamycin/disc), OFX10 (10 μg ofloxacin/ disc), AMC30 (20 μg amoxicillin + 10 μg clavulanic asit/disc), KF30 (30 μg cephalothin/ disc), TE30 (30 μg tetracycline/disc), AZM15 (15 μg azithromycin/disc), AMP20 (20 μg Amphotericin B/disc) were used as positive reference standards to determine the sensitivity of one strain/isolate in each microbial species tested. The inoculated plates were incubated for 24 h at 37ºC for mesophilic bacterial strains, 48 h for yeast, and 72 h for fungi isolates. Psychrotrophic bacteria were incubated at 20ºC for 48 h (14). The antimicrobial activity was evaluated by measuring the zone of inhibition against the test organisms. Each assay in the experiment was repeated twice. 

The experiment was a completely randomized design with four replications. The data was subjected to analysis of variance testing (ANOVA) and the means were separated by the Duncan multiple range test with a p*-*value of < 0.05 being significant.

## Results and Discussion

*Antioxidant activity and the total phenolic content*

**Figure 1:**
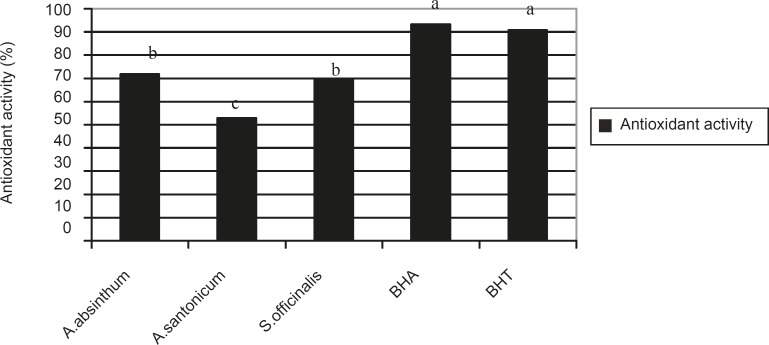
Antioxidant activity in *Artemisia absinthum, Artemisia santonicum a*nd *Saponaria offýcinalis*, BHA and BHT. **The a, b, c are the result of statistical analysis and show that there are significant differences among the plant species, BHA and BHT with regards to antioxidant activity where a p-value of < 0.05 was significant

 The antioxidant activity of *Artemisia absinthum, Artemisia santonicum *and *Saponaria officinalis *are given in Figure 1. There are statistical differences among the BHA, BHT and plant extracts in terms of antioxidant activity. The antioxidant activity was highest in standard BHA (200 mg/L) (measuring 93.21%) followed by BHT (200 mg/L) (90.71%), *Artemisia absinthum *(71.78%), *Saponaria officinalis *(70.00%) and* Artemisia santonicum *(62.86%), respectively. In other words the antioxidant activity decreased in the following order: BHA > BHT > *Artemisia absinthum *> *Saponaria officinalis *> *Artemisia santonicum*. 

The antioxidant activities of these 3 species can be attributed to the absence of some components that have antioxidant activity. The plant extracts nevertheless consist of various constituents. Therefore, determination of the componenets responsible for activity is very difficult. In a previous study, it was reported that the essential oils of *Artemisia dracunculus *exhibited antioxidant activity (16) supporting our findings.

**Figure 2:**
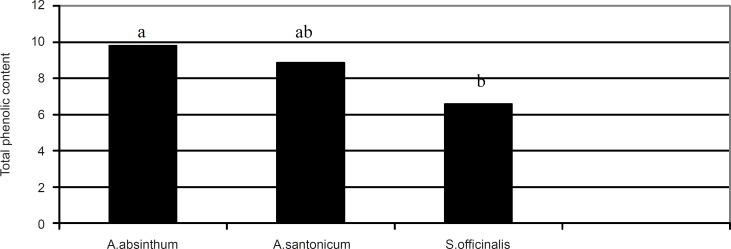
Total phenolic content (μg GAE/mg) of *Artemisia absinthum, Artemisia santonicum *and *Saponaria officinalis*. **The a, b or ab are the result of statistical analysis and show that there are significant differences among the plant species with regards to total phenolic content where p < 0.05 was statistically significant

The total phenolic content in the studied plants are illustrated in Figure 2. 

The highest total phenolic content was observed in *Artemisia absinthum *(9.79 μg GAE/mg) followed by *Artemisia santonicum *(15.38 μg GAE/mg) and *Saponaria officinalis *(6.57 μg GAE/mg), respectively. From the results shown in Figure 2, it can be seen that there is statistical difference between the plant extracts in terms of their total phenolic content (p < 0.05). In addition, there was a positive correlation (R = 0.819) between the total phenolic content and antioxidant activity in the plant samples. Several studies have reported the relationship between phenolic content and antioxidant activity. Some authors have found a correlation between the phenolic content and antioxidant activity, while others found no such relationship. Velioglu *et al*., (17) reported a strong relationship between the total phenolic content and antioxidant activity in certain plant products. There is a need to characterise phenolic compounds present within each plant extract to assign different antioxidant activities, to ascertain whether the phenolic structure affects antioxidant activity and also to determine whether synergism definitely occurs among certain phenolic compounds.

*Antimicrobial activity*

The antibacterial activity of *Artemisia absinthum, Artemisia santonicum *and *Saponaria officinalis *against a number of bacteria is shown in Table 1. 

**Table 1:** Antimicrobial activities of A*rtemisia absinthum, Artemisia santonicum *and S*aponaria officinalis*.

**Microorganisms**	***Artemisia absinthum***		***Artemisia santonicum***		***Saponaria officinalis***		**Positive control** ^a^
	**Aqueous**	**Methanolic**	**Aqueous**	**Methanolic**	**Aqueous**	**Methanolic**	
*Bacillus subtilis *ATCC 6633	-	19	-	18	-	10	17 (NV 30)
*Staphylococcus aureus *ATCC 29213	12	15	12	18	-	18	20 (SCF 105)
*Streptococcus pyogenes *ATCC 176	-	11	-	12	-	-	30 (NV5)
*Salmonella typmiruim *RSSK 95091	-	13	-	13	-	12	9 (OFX 10)
*Saccharomyces cereviciae *6541	-	9	-	18	-	-	8 (AMP 20)
*Bacillus cereus *6230	-	13	-	19	-	-	12 (CC 2)
*Candida albicans *ATCC 1223	-	9	-	6	-	10	15 (AMP 20)
*Streptococcus thermophilus *6453	-	15	8	18	9	10	10 (OFX 10)
*Pseudomonas aeruginosa *ATCC 9027	-	17	10	23	-	19	36 (TE 30)
*Klebsiella pneumonia *subsp. *pneumonia *2124	8	8	-	6	-	8	13 (OFX 10)
*Staphylococcus hominis *3221	7	17	9	21	-	23	24 (KF 30)
*Enterobacter cloaceae *7418	-	8	-	9	--	7	10 (OFX 10)
*Escherichia coli *1328	7	11	-	10	-	8	18 (CC 2)
*Proteus mirabilis *3242	-	10	-	8	-	7	12 (OFX 10)
*Klebsiella pneumonia *subsp. *ozanae *5713	-	6	-	8	-	6	19 (CC 2)
*Providencia alcaliaciens *3215	11	14	-	13	-	12	10 (AZM)
*Acinetobacter lwoffi *2819	-	11	7	14	10	11	17 (AMC 30)
*Pseudomonas pseudoalcaligenes *3445	-	6	-	8	-	-	24 (OFX 10)
*Pseudomonas fluorescens *7324	9	6	-	8	-	6	31 (OFX 10)
*Pseudomonas putida *1617	-	16	7	18	7	22	16 (TE 30)
*Flavobacterium indologenes *1520	-	6	-	8	11	-	27 (AZM 15)
*Yersinia enterocolitica *0184	11	6	-	8	7	6	26 (AZM 15)
*Alcaligenes feacalis *0452	-	-	-	12	-	-	7 (SAM 20)
*Penicillium brevicompactum*	-	9	-	7	-	8	14 (AMP 20)
*Trichothecium roseum*	-	11	-	16	-	-	21 (AMP 20)
*Aspergillus niger*	-	-	-	7	-	-	21 (AMP 20)

^a^ SCF105 (30 μg sulbactam + 75 μg cefoperazona/disc), NV30 (30 μg novobiocin/disc), NV5 (5 μg novobiocin/disc), SAM20 (10 μg sulbactam + 10 μg ampicillin/disc), CC2 (2 μg clindamycin/disc), OFX10 (10 μg Ofloxacin/disc), AMC30 (20 μg amoxicillin + 10 μg clavulanic asit/disc), KF30 (30 μg cephalothin/disc), TE30 (30 μg tetracycline/disc), AZM15 (15 μg azithromycin/disc), AMP (20 μg/disk Amphotericin) were used as positive reference standards antibiotic discs (oxoid

As shown in Table 1, the methanol extracts of the plants samples were found to be more effective on microorganisms in comparison to the aqueous extracts. The methanolic extract of *Artemisia santonicum *exhibited antibacterial activity against all investigated microorganisms with a 6-18mm zone of inhibition*. *The *Artemisia absinthum *extract also showed antibacterial activity against all tested microorganisms (6-19 mm inhibition zone), apart from *Alcaligenes feacalis *and *Aspergillus niger *(Table 1). On the other hand, *Saponaria officinalis *had antioxidant activity against 19 out of 27 microorganisms (6-23 mm inhibition zone)*. *However, aqueous extracts of *Artemisia absinthum, Artemisia santonicum *and *Saponaria officinalis *showed only weak antimicrobial activity against 7 out of 27 (7-12 mm inhibition zone), 7 out of 27 (7-12 mm inhibition zone) and 5 out of 27 (7-11 mm inhibition zone) microorganisms investigated, respectively (Table 1). Previously, the antifungal and antibacterial effects of the *Artemisia *species have been reported mostly based on bacteria of clinical origin (16).

Intersestingly the inhibition effects of the methanol extracts of *Artemisia absinthum *and *Artemisia santonicum *against some microorganisms such as *Bacillus subtilis, Salmonella typmiruim, Saccharomyces cereviciae, Bacillus cereus, Streptococcus thermophilus, Providencia alcaliaciens *and* Pseudomonas putida *were higher than that of the positive controls (Table 1). The results obtained during the course of the present study are in agreement to a certain extent with the traditional uses of *Artemisia absinthum,* *Artemisia santonicum *and *Saponaria officinalis* in particular within the Eastern Anatolia region of Turkey.

To conclude, the methanolic extracts of the three plant species found in the Eastern part of Turkey were found to possess phenolics in addition to exhibiting antioxidant activity. The results achieved using these assays provides simple data making it possible to classify extracts according to their total phenolic content and antioxidant potential. The therapeutic value of the plant extracts may be partly due to their antioxidant activity. Further studies on the absorption and the effects of phytochemicals present in the plant extracts on antioxidant status in animal models are needed to evaluate their potential health benefits. Based on these results, it is possible to conclude that the aerial parts of *Artemisia absinthum, Artemisia santonicum *and *Saponaria officinalis *exhibit antibacterial activity against a number of bacteria.
